# DENSEN: a convolutional neural network for estimating chronological ages from panoramic radiographs

**DOI:** 10.1186/s12859-022-04935-0

**Published:** 2022-10-14

**Authors:** Xuedong Wang, Yanle Liu, Xinyao Miao, Yin Chen, Xiao Cao, Yuchen Zhang, Shuaicheng Li, Qin Zhou

**Affiliations:** 1 The Clinical Research Center of Shaanxi Province for Dental and Maxillofacial Diseases and Department of Implant Dentistry, College of Stomatology, Xi’an Jiaotong University, Xi’an, 710004 People’s Republic of China; 2 The Byoryn Technology Co., Ltd, Shenzhen, 518122 China; 3 School of Forensic and Medicine, Xi’an Jiaotong University Xi’an, Xi’an, 710004 Shaanxi People’s Republic of China; 4 City University of Hong Kong, 83 Tat Chee Ave, Kowloon Tong, Hong Kong China; 5 The BGI Education Center, University of Chinese Academy of Sciences, Shenzhen, 518083 China

**Keywords:** Soft Stagewise Regression Network, Chronological age estimation, Forensic anthropology, Orthopantomogram

## Abstract

**Background:**

Age estimation from panoramic radiographs is a fundamental task in forensic sciences. Previous age assessment studies mainly focused on juvenile rather than elderly populations (> 25 years old). Most proposed studies were statistical or scoring-based, requiring wet-lab experiments and professional skills, and suffering from low reliability.

**Result:**

Based on Soft Stagewise Regression Network (SSR-Net), we developed DENSEN to estimate the chronological age for both juvenile and older adults, based on their orthopantomograms (OPTs, also known as orthopantomographs, pantomograms, or panoramic radiographs). We collected 1903 clinical panoramic radiographs of individuals between 3 and 85 years old to train and validate the model. We evaluated the model by the mean absolute error (MAE) between the estimated age and ground truth. For different age groups, 3–11 (children), 12–18 (teens), 19–25 (young adults), and 25+ (adults), DENSEN produced MAEs as 0.6885, 0.7615, 1.3502, and 2.8770, respectively. Our results imply that the model works in situations where genders are unknown. Moreover, DENSEN has lower errors for the adult group (> 25 years) than other methods. The proposed model is memory compact, consuming about 1.0 MB of memory overhead.

**Conclusions:**

We introduced a novel deep learning approach DENSEN to estimate a subject’s age from a panoramic radiograph for the first time. Our approach required less laboratory work compared with existing methods. The package we developed is an open-source tool and applies to all different age groups.

## Background

Chronological age estimation presents a key feature in forensic anthropology and evidence, especially in criminal investigations or disasters [8, 16, 19, 38, 55, 56, 64, 66]. Despite the rapid advance of DNA sequencing technologies [41], Age determination with DNA methods is nontrivial. Due to higher juvenile criminals and diverse reduced or full age of criminal responsibility around different legal systems, children and teenagers’ age estimation through morphological methods is insufficient [10, 61].

Several studies focus on the relationship between epiphyseal closure and chronological age [21]. There are many factors related to epiphyseal fusion, such as gender, genetics (race), and geography [15, 57]. Due to the uncompleted skeletal development, the bone age assessment method is merely used to estimate immature individuals [25, 46]. Developmental skull sutures are unreliable indicators for adult age estimation due to inaccuracies and unstable.

Dental tissues may provide vital clues in identifying an unknown deceased person in the field of archeology, paleoanthropology, and forensics [27, 45, 48, 50, 52, 59]. As the most rigid tissues in the human body, teeth resist more chemical and mechanical stress and can be preserved under variable environments for a long time [20, 43]. Many morphological methods based on tooth changes, such as amino acid racemization, are time-consuming and destructive and neglect the considerations of topology, anthropogenic, and geological evolution [27]. Non-invasive clinical and radiological examinations of dental issues are universal methods for chronological age estimation [62].

Some studies estimated the ages involving weighty medical examinations, relying on statistical methods [13, 36, 44, 66]. Demirjian estimated the chronological age of the seventh tooth from the left side of the mandible. Moores proposed a way to estimate the age with 14 stages of mineralization for developing single or multi-rooted teeth [13]. However, these methods are relatively time-consuming and rely on manual processes whose results are affected by the observer subjectivity.

With the continuous development of data science, deep learning has been applied to clinical medicine and imaging research, achieving comparable or higher accuracy than practitioners’. Currently, some studies have used deep learning methods for pediatric estimation by analyzing the hand and wrist bone radiographs [3, 12, 22, 24, 26, 35, 36, 40, 60, 65].

Some studies have also applied deep learning research [1, 9, 11, 33, 44, 47, 53, 63]. (1) Walter de Back estimated age with Bayesian CNNs and makes a satisfactory result. With the dataset covering individuals from 5 to 25 years old, the proposed model resulted in higher MAE for the 22–25 years old group [12]. (2) Jaeyoung Kim [30] employed a curriculum learning strategy in developing an automatic chronological age estimation system for all age groups using panoramic dental X-ray. The performance of the model in group 19+ was still unsatisfying, with MAE 4.398. (3) Another deep learning method, DANet, proposed in the latest study, is unsuitable for the elderly population [63]. The author claimed that the performance of the network deteriorated when the experiment was expanded to include older participants. It is difficult to assess age accurately when people are older than 25 because permanent teeth will completely be formed during this time [51]. These algorithms are the latest methods and the state-of-the-art methods on OPT processing at present.

We proposed DENSEN; a regression model derived from Soft Stagewise Regression Network (SSR-Net) for age estimation of panoramic radiographs. We used CNNs to establish an automated and accurate forensic age assessment approach. For verifying the reliability of the model, we also used other CNN-based methods that have reported outstanding performance in the latest computer vision research to compare.

## Results

We adopt mean absolute error (MAE) as a criterion to evaluate the model. Below *x* and *y* are *D* dimensional vectors, and $x_i$ denotes the value on the *i*-th dimension of *x*. *D* is the number of samples, *x* is the predicted age, and *y* is the ground truth age.

1 $$\begin{aligned} L= \sum _{i=1}^{D}|x_i-y_i| \end{aligned}$$

Most published methods trained gender-specific models to improve accuracy. However, in many scenarios, the individuals’ gender is unknown. To address this issue, we omitted sex-related features in our model. To explore the predictors’ performance at different ages, we divided the test set into four age groups 3–11 (children), 12–18 (teens), 19–25 (young adults), and 25+ (adults), respectively. Due to the research was conducted in Hong Kong, we chose 11 and 18 as age boundaries in accordance with the local laws of Hong Kong. Relevant provisions are shown on the official website of the government (https://www.smartid.gov.hk/en/Replacement-arrangement-for-Children-holding-old-form-of-smart-identity-cards/index.html)

### Performance of DENSEN

**Fig. 1:**
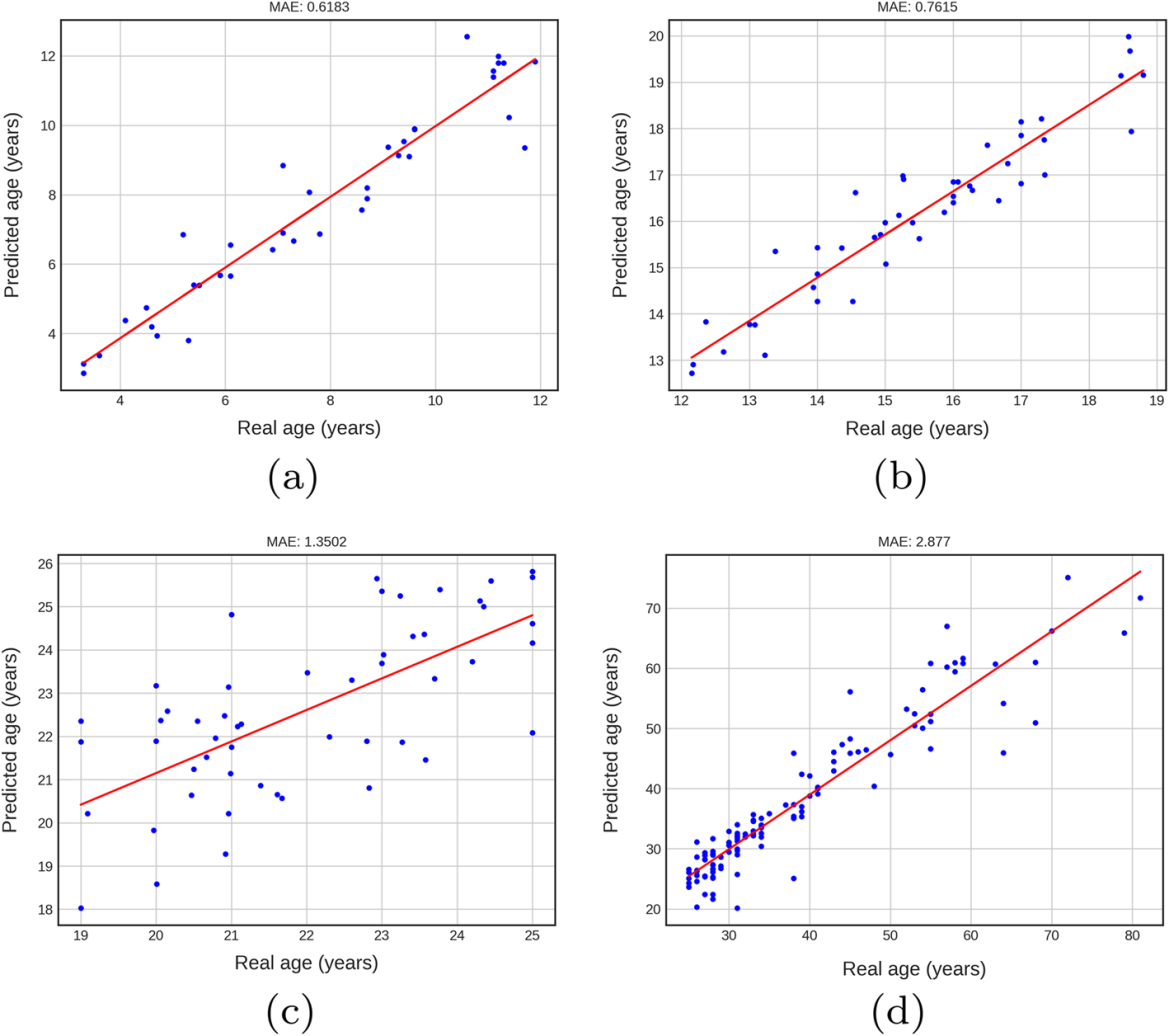
Performance of DENSEN in children, teens, young adults and adults groups.** a**–**d** display the performance of the DENSEN for the four age groups. The figures show that the predictor works ideally in children and teens groups with MAEs of 0.6885 and 0.7615, respectively. MAEs increase in the young adult and adult groups to MAE 1.3502 and 2.8770, respectively

Figure 1a–d display the performance of the DENSEN for the four age groups. The figures show that the predictor works ideally in children and teens groups with MAEs 0.6885 and 0.7615, respectively. However, MAEs increase in the young adult and adult groups to MAE 1.3502 and 2.8770, respectively. It is challenging to predict age for the elder groups, especially in the adult group, because their permanent teeth are fully formed, leaving fewer features extracted.

### Performance of Bayesian CNNs Net and DANet

**Fig. 2:**
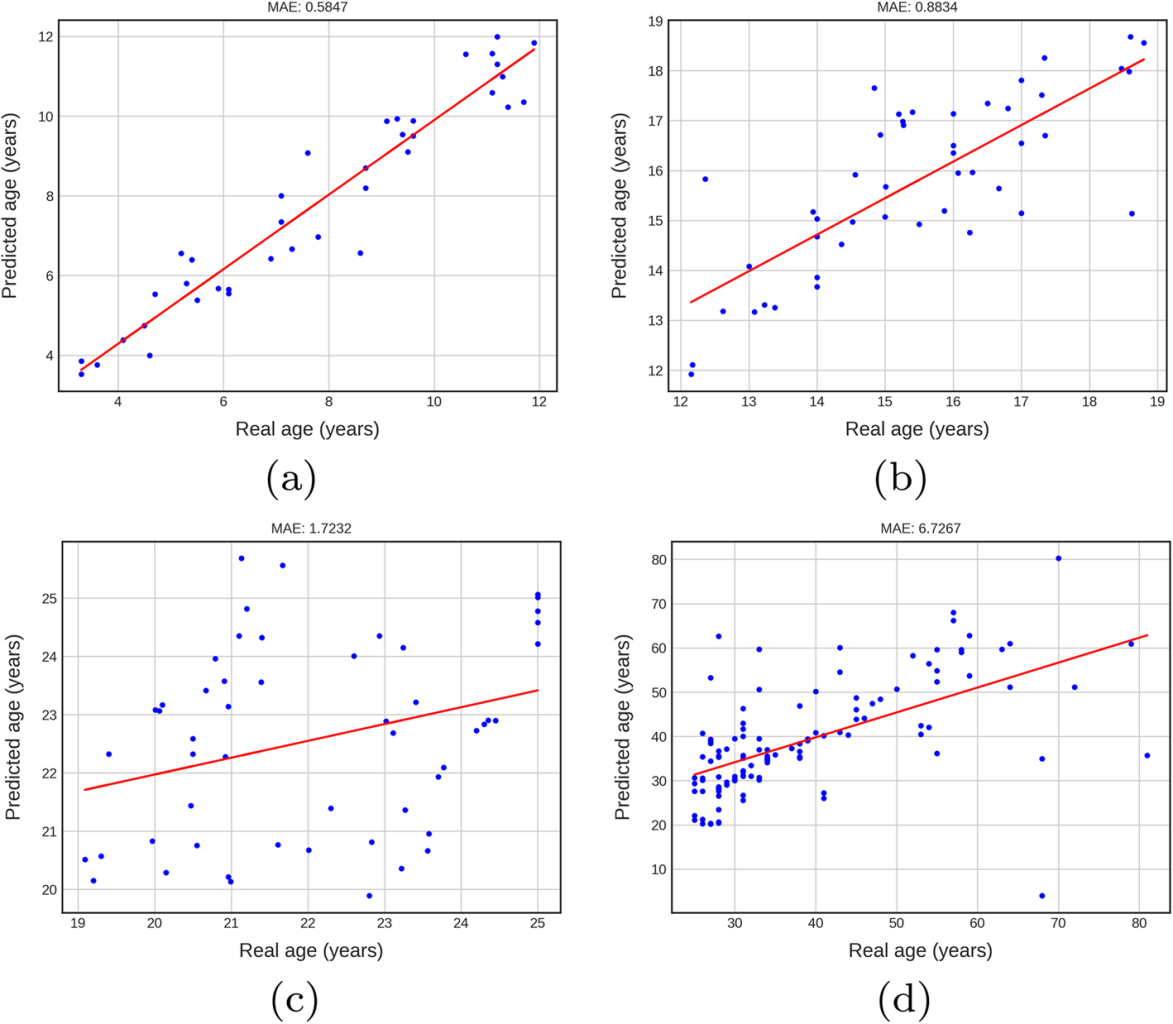
Performance of Bayesian CNN in children, teens, young adults and adults groups.** a**–**d** show the performance of Bayesian CNN Net in these four groups.** a** suggests that there is generally a high correlation between the ground truth ages and predicted ones in the children group with MAE 0.5847. In the teen group, there is a slight increase in MAE (MAE = 0.8834).** c** and** d** present the model with difficulty handling the age estimation in young adults (MAE = 1.7232) and adult (MAE = 6.7267) groups

Figure 2a–d show the performance of Bayesian CNN Net in these four groups. The Fig. 2a suggests that there is generally a high correlation between the ground truth ages and predicted ones in the children group with MAE 0.5847. In the teens group, there is a slight increase in MAE (MAE = 0.8834). Figure 2c, d present the model with difficulty handling the age estimation in young adults (MAE = 1.7232) and adult (MAE = 6.7267) groups.

**Fig. 3:**
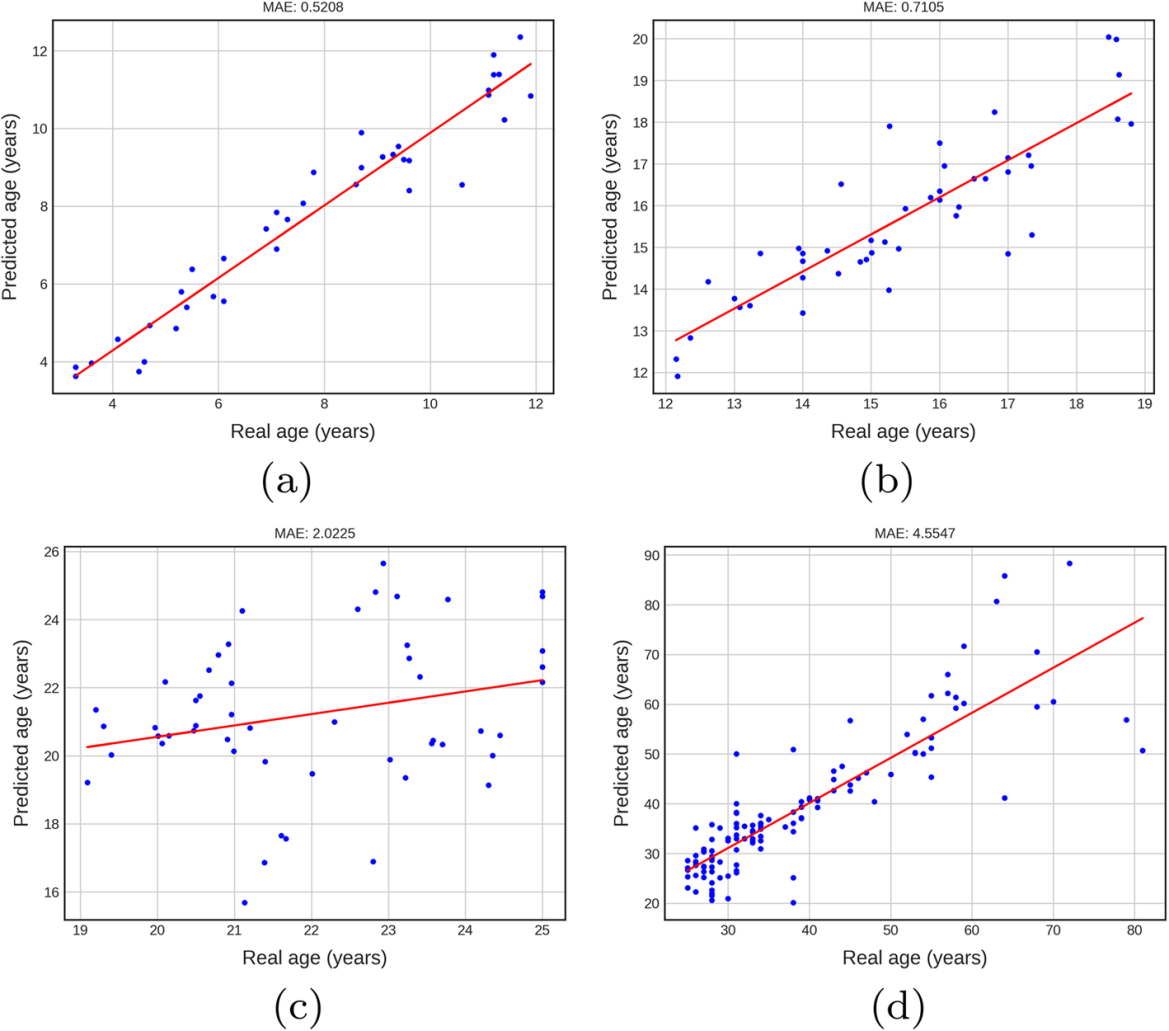
Performance of DANet in children, teens, young adults and adults groups.** a**–**d** present DANet performance in the four age groups. The MAEs generated by the DANet are 0.5208, 0.7105, 2.0225, and 4.5547 for children, teens, young adults, and adult groups, respectively. The results display that the model estimates age accurately in the children group and teen group. However, in the young adult and adult groups, the model works with unfitted MAE 2.0225 and 4.5547, respectively

Figure 3a–d present DANet performance in the four age groups. The MAEs generated by the DANet are 0.5208, 0.7105, 2.0225, 4.5547 for children, teens, young adults, and adult groups, respectively. The results display that the model estimates age accurately in the children group and teen group. However, in the young adult and adult groups, the model works with unfitted MAE 2.0225 and 4.5547, respectively.

### Comparison and further exploration

The statistical results of the three CNN networks applied in our work are listed in Table 1. As presented in Table 1, there is a similar trend that these three networks perform relatively accurately in the early stage. DENSEN is comparable with other related CNN methods (mentioned in Methods 2.3) with MAE 0.6183, 0.7615, 1.3502, and 2.8770 in the four age groups, respectively.

**Table 1:** Performance comparison between DENSEN with state-of-the-art approaches

Age group (yo)	3–11 (children)	12–18 (teens)	19–25 (young adults)	25+ (adults)	Model size (MB)
DENSEN	0.6885	0.7615	1.3502	2.8770	1.0
Bayesian CNNs Net	0.5847	0.8834	1.7232	6.7267	22.8
DANet	0.5208	0.7105	2.0225	4.5547	97.6

Compared with the other two CNN methods, DENSEN demonstrates higher accuracy (MAE = 2.8770) in the adult group. Besides, the model size generated by DENSEN takes only 1.0 MB memory, less than the other two models. DENSEN is more competitive to be applied to devices that have limited memory space.

Besides,we supplemented the baseline experiments with several machine learning methods and the results are presented in Additional file 1.

**Fig. 4:**
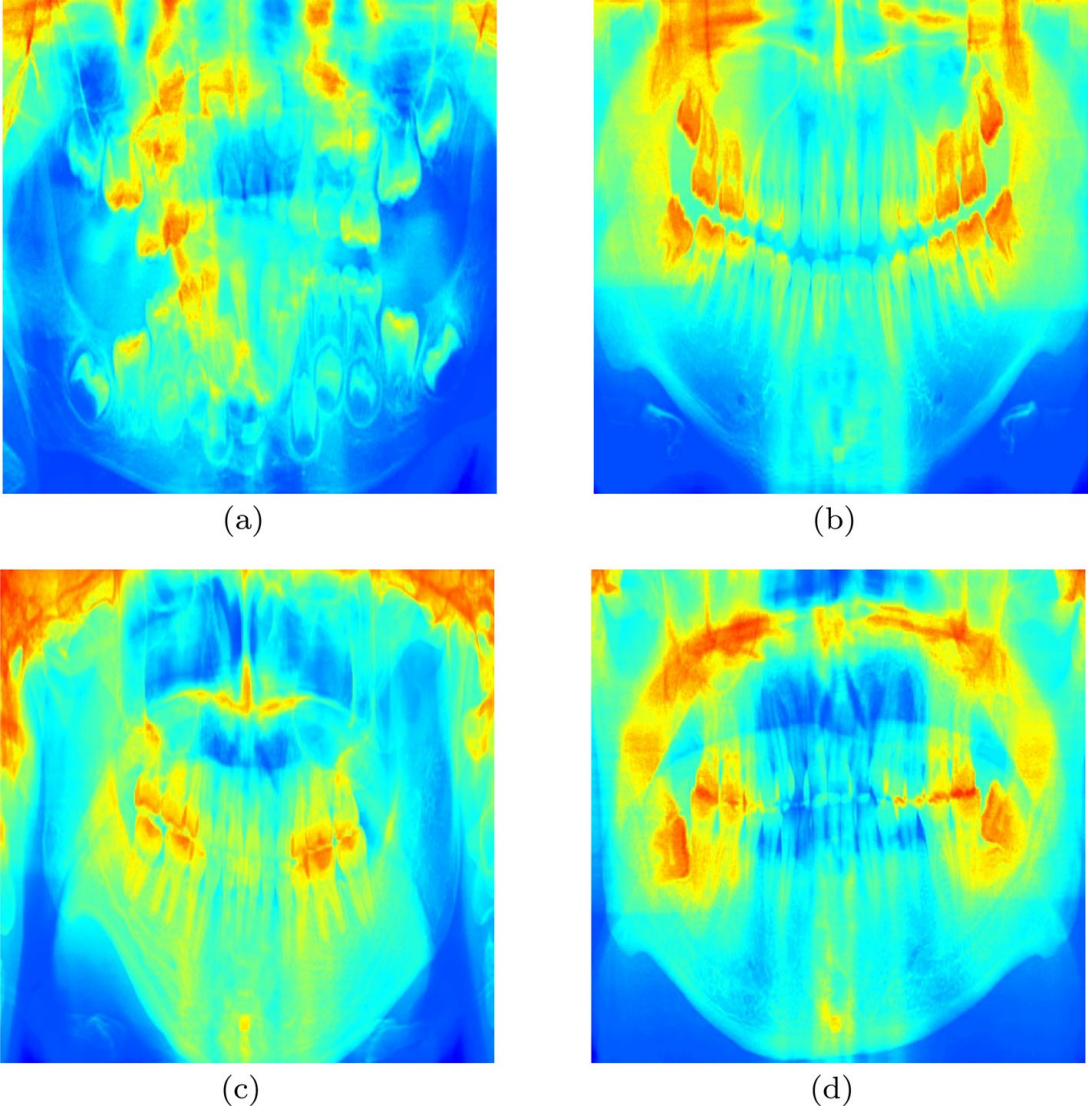
Saliency maps of children, teens, young adults and adults groups. We randomly chose images within the four groups. Then we feed the images into DENSEN. After calculating the last linear layer’s gradient, we feed the photos into a previous convolutional layer to visualize the saliency map’s learned features

To further explore the deep learning method’s effect in tackling the age estimation problem, we analyzed the saliency map to detect the focused image region, as presented in Fig. 4a–d. Firstly, we randomly chose images within the four groups. Then we feed the images into DENSEN. Secondly, after calculating the last linear layer’s gradient, we feed the photos into a previous convolutional layer to visualize the saliency map’s learned features. Finally, we observed that the red region provides more features to the model than the blue region. The most formative regions in these maps are located in the teeth area. The molars contribute the most in the elder groups. Unexpectedly, the maxillary sinus and nasal septum regions are labeled as markers in the model.

## Discussion

According to the FBI records, as of 2020, there were 365,348 National Crime Information Center entries for missing children in the United States [4]. Many social services, such as the AMBER Alert system in the US and Canada, provide up-to-date information searching for missing vulnerable children [18].

Some studies used multiple technologies, including artificial intelligence and digital image processing, to create age-progression photos. Nevertheless, some studies implied that age progressions do not benefit more [2, 6, 28, 29, 32, 39, 34]. Recent findings estimated that over 30% of the world’s children haven’t registered or registered with doubted age documentation [49]. Thus, estimation of an individual’s age has an essential value in establishing an anthropological profile.

Forensic dentistry has great applicability in forensic identification by studying specific characteristics. Chronological age estimation from OPTs is of vital importance in criminal investigations or disasters. For most areas globally, OPT might be an appropriate way because it is rapidly cost-effective and convenient [5]. However, existing methods mainly focused on estimating juvenile rather than elderly adults (>25years old). Most studies were based on statistical or scoring-based methods, requiring wet-lab experiments and prior professional knowledge.

Currently, the utilization of smartphones in clinical and biological research is increasing. Joonchul Shin developed a Smart Forensic Phone system for estimating age from bloodstain in 2017 [58]. Khurram proposed a software named Deepgender for smartphones to classify gender in 2019 [23]. Our results implied that the DENSEN method might be competitive to utilize memory shortage devices than other CNN Net due to its compact. Based on the smaller model size, our model could apply to memory-limited devices. Besides, we performed several deep learning networks to tackle the chronological age estimation problem. We successfully applied the CNN to estimate the ages relatively accurately using a DENSEN. Our study is the first use of DENSEN to predict age estimation from dental images.

The DENSEN is inspired by SSR-Net and DEX. Age estimation is recast as a multi-class classification issue in DEX, and the classification results are transformed into regression by computing the predicted value as the age. Additionally, we adopt the double stream approaches motivated by the complementary 2-stream structure proposed by Yang et al. [67]. The difference between these two streams is the activation function (RELU vs. Tanh) and the difference in pooling (average vs. maximum). Feature fusion will be performed on the dual-stream output between each stage.

We still need to improve the age assessment system’s accuracy, especially in young adults and adults. (1) Firstly, our next objective is to enlarge the size of origin dental X-ray images and control the quality of the images. (2) Secondly, we will package our dental network into mobile applications in the future that enables forensic to complete the identification work conveniently. At present, gender characteristics are not considered in our complete process design. The main reason is that some of the medical images we collected have unknown gender labels. In the process of actual forensic identification, appraisers often encounter cases with unknown gender. Our method can solve this problem well. We will collect more gender-specific data in the next iteration and provide an interface for gender characteristics so that the DENSEN can be compatible with a broader range of application scenarios. (3) Thirdly, the unexpected regions in the saliency maps also worth further study. Then we will check if these regions match with the dental doctor’s opinions. We speculate that these regions can be used in a particular age group as labeled markers to improve the model’s accuracy.

## Conclusions

We introduced a novel deep learning approach DENSEN to estimate a subject’s age from a panoramicradiograph for the first time. In comparison to current techniques, the DENSEN needs less laboratory time and it is an open-source model that can be adopted by people of all ages groups. The DENSEN approach estimated age accurately and efficiently from dental X-ray images at first and is useful in lightweight forensic applications.

## Methods

### Data preprocessing

In this work, the orthopantomograms (OPTs) are from the Clinical Research Center of Shaanxi Province for Dental and Maxillofacial Diseases & Department of Implant Dentistry, College of Stomatology, Xi’an Jiaotong University. OPT is also known as an orthopantomograph, pantomogram, or panoramic radiograph[31].

All images were anonymized to protect the confidential information of the participants. All photos and experiments involved were in line with the institutional and national research committees’ ethical standards and the Helsinki Declaration.

Limited by the sampling source, we manually removed severely distorted images. These lesions and deformities would introduce pathological noise and cover the target information in the image itself. After quality control of the images, we have 1,903 valid dental photos left. We randomly divided the images into the training set and the test set with a random size of 0.865, which results in 1647 images and 256 images, respectively.

**Fig. 5:**
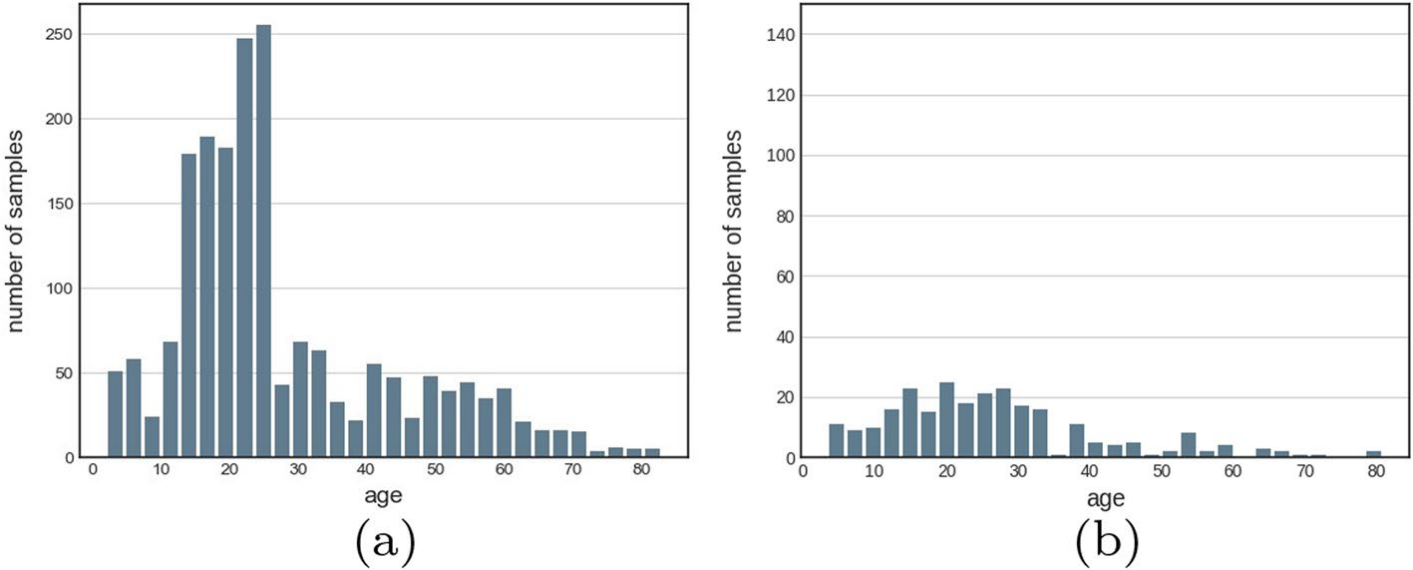
Number of participants according to ages in train set (**a**) and test set (**b**)

Table 2 lists the distribution of the images according to gender. Figure 5a, b show that the data unevenly distributes according to the age both on the training set and test set.

**Table 2:** The gender distribution

	Male	Female	Unknown	Total
Train	882	750	15	1647
Test	122	132	2	256
Total	1004	882	17	1903

For preprocessing of OPTs, we used data augmentation to save the computing resources and improve the training set. Data augmentation effectively improves the model’s robustness, generating new data through some transformation based on training data. Using augmentation, we enlarged the size of the training set to improve our model’s generalization.

We adopt two methods to enhance our dental OPTs, filtering augmentation and sliding augmentation. Filtering augmentation mainly uses sharpening, passivization, and edge hardening against different image quality levels. Sliding augmentation reduces the impact of critical elements’ location in the input image.

**Fig. 6:**
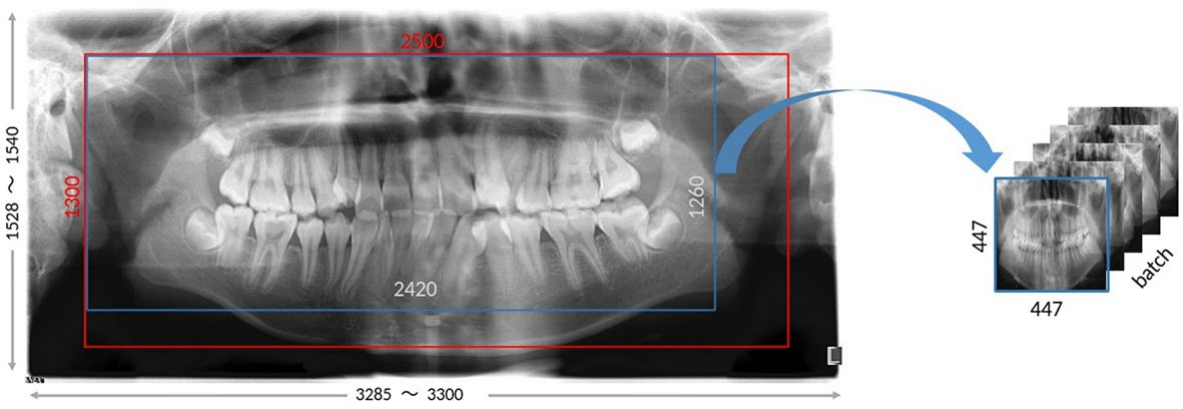
Sliding augmentation

The Fig. 6 displays the visualization results of sliding augmentation. Table 3 presents the detailed factors used in our case, including resizing pixels, rotating the images in different degrees, translating the coordinates of x and y, zooming the image in size from 0.9 to 1.1, and changing the brightness of images.

**Table 3:** Data augmentation transformations used to improve the training set

Transformation	Factor
Resize	(447, 447) pixels
Rotation	(− 1, 1) degrees
Translation X	(− 5, 5) pixels
Translation Y	(− 5, 5) pixels
Zoom	(0.9, 1.1)
Brightness	(0.5, 1.0)

### Multi-classes regression network for DENSEN

We employed a CNN network DENSEN derived from SSR-Net [40]. DENSEN produced a lightweight model size related to portable devices. DENSEN and SSR-Net are both inspired from DEX [7, 14, 37, 42, 54]. Age estimation is recast as a multi-class classification issue in DEX, and the classification results are transformed into regression by computing the predicted value as the age. One disadvantage of DEX is that it necessitates a huge number of neurons, one for each age class. The product of the number of features and the number of neurons determines the number of connections in the final fully connected layer.

DENSEN performed multiclasses regression to address the age estimation issue and then turned the classification results into regression by calculating the expected values. The network adopts a coarse-to-fine strategy and divides the classification into multiple stages. Each of the stages monitors the decision of the previous stage for a better evaluation of age. Meanwhile, each stage consists of a few classes and requires few neurons. Moreover, DENSEN adopts the dynamic range to address the quantization problems of the age.

#### Stagewise Regression

We divided all age datasets into $\mathrm {K}$ stages uniformly.

Let $s_{k}$ as the number of bins for the $\mathrm {k}$-th stage. At each stage, DENSEN trains a network $\mathrm {F}_{k}$ that produces the distribution $\overrightarrow{\mathrm {p}}^{(\mathrm {k})}=$ $\left( \mathrm {p}_{0}^{(\mathrm {k})}, \mathrm {p}_{1}^{(\mathrm {k})}, \ldots , \mathrm {p}_{s_{k}-1}^{(\mathrm {k})}\right)$ and the then age $(\tilde{\mathrm {y}})$ prediction could be formulated as follows for stagewise regression [67].

2 $$\begin{aligned} \tilde{y}=\sum _{k=1}^{K} \vec {p}^{(k)} \cdot \vec {\mu }^{(k)}=\sum _{k=1}^{K} \sum _{i=0}^{s_{k}-1} p_{i}^{(k)} \cdot i\left( \frac{V}{\prod _{j=1}^{k} s_{j}}\right) \end{aligned}$$

We set three stages (K=3) in total. There are three bins for either stage ($s_{1}=s_{2}=s_{3}=3$). Stage one classifies the dental X-ray image as youth (0-27), middle age (28-54), and old age (55-81). For stage two, each bin from stage one is further subdivided into $s_{2}=3$ bins. The rest is inferred by analogy. Thus, the width of the bins in stage three is $81/27 = 3$. The advantage of stagewise regression is that the number of classes is small at each stage. The small number of classes led to much fewer parameters and built a more compact model. The input-dependent dynamic range provides more accurate refinement according to the input image [67].

#### Network Architecture

**Fig. 7:**
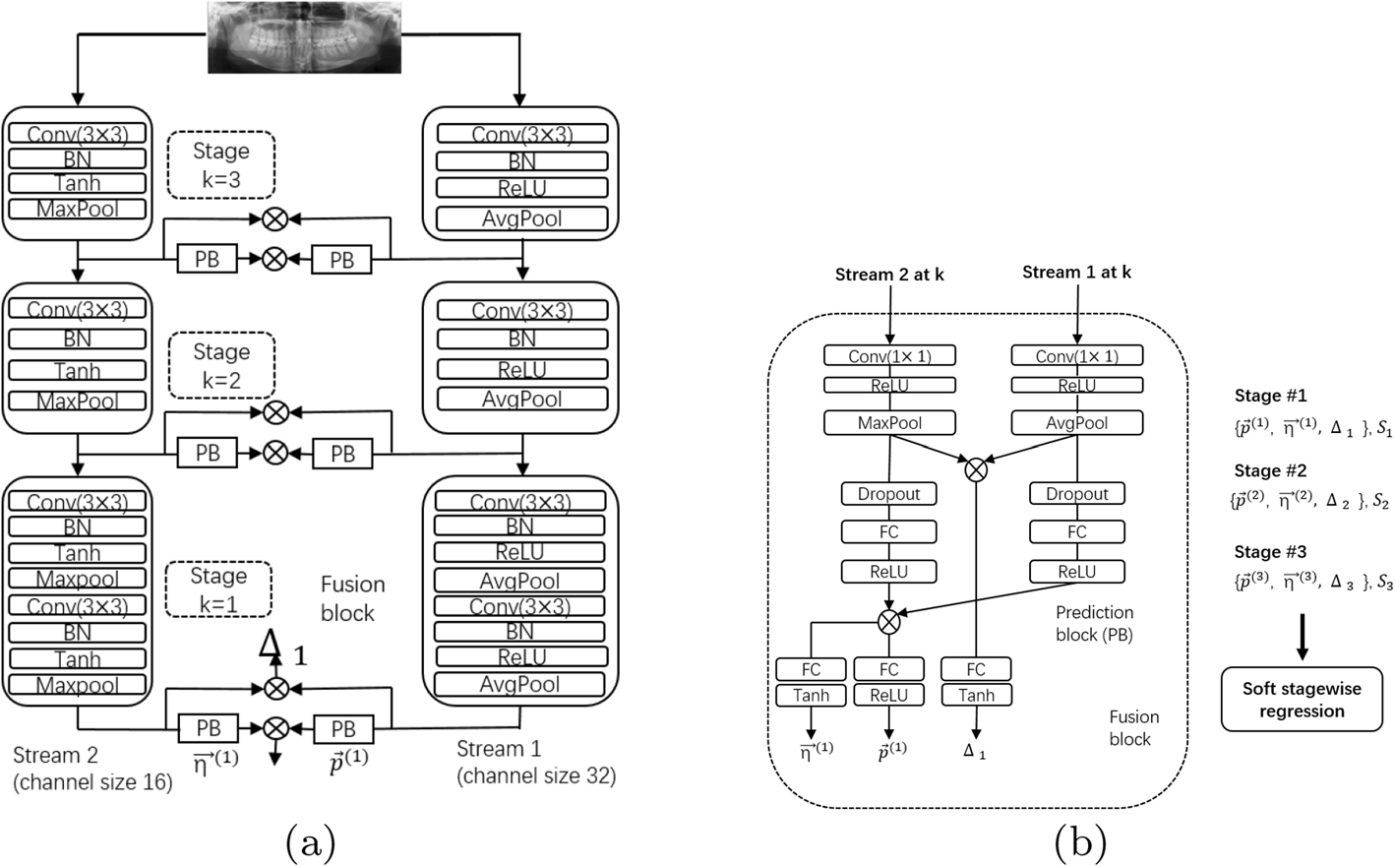
Structure of DENSEN.** a** shows that the network adopts a 2-stream model similar to its initial network architecture. There are two heterogeneous streams. For both streams, the basic building block is composed of convolution layers, batch normalization, non-linear activation, and pooling layers.** b** presents different activation functions (ReLU versus Tanh) and pooling (average versus maximum) adopted for each stream

Figure 7a, b illustrate the overall network architecture of the DENSEN.

We also adopt a 2-stream model the same as its initial network architecture. There are two heterogeneous streams. For both streams, the basic building block is composed of $3 \times 3$ convolution, batch normalization, non-linear activation, and $2 \times 2$ pooling layers. Different activation functions (ReLU versus Tanh) and pooling (average versus maximum) are adopted for each stream to heterogeneous.

### Related CNN networks

Bayesian CNN Net and DANet are related CNN-methods in the field of image processing. Here, we adopt these two CNN networks to compare with our DENSEN.

#### Bayesian CNNs Net

Bayesian CNNs Net offers better robustness against over-fitting on small data than traditional approaches [17]. Walter de Back applied Bayesian CNNs to automated forensic age estimation based on dental X-ray images. The resulting model predicted the age group from 4 to 7 with an MAE of about 1.0 [12]. Due to the shortage of data sources, the dataset covers from 5 to 25 years old. Thus, we applied our relatively more comprehensive range datasets to this network.

#### DANet

With more complex architecture, DANet consists of a sequential CNN to predict age. The network was used in the latest research to estimate chronological ages from X-ray images [63]. The model makes accurate predictions in young subjects with an MAE of only 0.6 [63]. Here we also attempt to apply our data to this network.

## Supplementary Information

**Additional file 1.** Performance of baseline machine learning methods.

## Data Availability

The data and code are available on https://delta.cs.cityu.edu.hk/xuedowang2/dental.

## Abbreviations

SSR-Net: Soft Stagewise Regression Network
OPT: Orthopantomogram
MAE: Mean absolute error
DNA: Deoxyribonucleic acid
CNN: Convolutional neural network
RELU: Rectified linear unit
Tanh: Hyperbolic tangent function
