# Correction: DENSEN: a convolutional neural network for estimating chronological ages from panoramic radiographs

**DOI:** 10.1186/s12859-022-05115-w

**Published:** 2022-12-22

**Authors:** Xuedong Wang, Yanle Liu, Xinyao Miao, Yin Chen, Xiao Cao, Yuchen Zhang, Shuaicheng Li, Qin Zhou

**Affiliations:** 1 The Clinical Research Center of Shaanxi Province for Dental and Maxillofacial Diseases and Department of Implant Dentistry, College of Stomatology, Xi’an Jiaotong University, Xi’an, 710004 People’s Republic of China; 2 The Byoryn Technology Co., Ltd, Shenzhen, 518122 China; 3 School of Forensic and Medicine, Xi’an Jiaotong University Xi’an, Xi’an, 710004 Shaanxi People’s Republic of China; 4 City University of Hong Kong, 83 Tat Chee Ave, Kowloon Tong, Hong Kong, China; 5 The BGI Education Center, University of Chinese Academy of Sciences, Shenzhen, 518083 China

**Correction: BMC Bioinformatics (2022) 23:426** **https://doi.org/10.1186/s12859-022-04935-0**

Following publication of the original article [1]. the authors flagged an error in the affiliations information of their article: affiliation ‘5’, which is only applicable for the fourth author, Yin Chen, had been assigned to all authors.

The article has now been corrected and the corrected affiliation information may be seen in this erratum. The authors thank you for reading and apologize for any inconvenience caused.

