# Supplementary material for: DENSEN: a convolutional neural network for estimating chronological ages from panoramic radiographs
Source: BMC Bioinformatics. 2022 Oct 14;23(Suppl 3):426. doi: 10.1186/s12859-022-04935-0 (PMC9569056; doi:10.1186/s12859-022-04935-0)
Supplement: Supplementary file 1 — Additional file 1. Performance of baseline machine learning methods. [file 12859_2022_4935_MOESM1_ESM.pdf]

Additional Files

Additional file 1 — Baseline results

Table 1 Performance of baseline meachine learning methods

Age group (yo)	3-11(children)	12-18(teens)	19-25(young adults)	25+(adults)	Model size (MB)
Linear Regression	1.7255	5.6684	4.7888	10.4752	135.6
Logistic Regression	4.2512	2.7375	5.7772	8.2185	77.4
Ridge Regression	3.8291	5.7335	8.9326	12.7332	89.6
Lasso Regression	2.4573	2.8532	4.7331	5.1351	112.5
